# Global integrated drought monitoring and prediction system

**DOI:** 10.1038/sdata.2014.1

**Published:** 2014-03-11

**Authors:** Zengchao Hao, Amir AghaKouchak, Navid Nakhjiri, Alireza Farahmand

**Affiliations:** 1 University of California, Irvine, E4130 Engineering Gateway Irvine, Irvine, CA 92697-2175, USA

**Keywords:** data integration, Global location, North America

## Abstract

Drought is by far the most costly natural disaster that can lead to widespread impacts, including water and food crises. Here we present data sets available from the Global Integrated Drought Monitoring and Prediction System (GIDMaPS), which provides drought information based on multiple drought indicators. The system provides meteorological and agricultural drought information based on multiple satellite-, and model-based precipitation and soil moisture data sets. GIDMaPS includes a near real-time monitoring component and a seasonal probabilistic prediction module. The data sets include historical drought severity data from the monitoring component, and probabilistic seasonal forecasts from the prediction module. The probabilistic forecasts provide essential information for early warning, taking preventive measures, and planning mitigation strategies. GIDMaPS data sets are a significant extension to current capabilities and data sets for global drought assessment and early warning. The presented data sets would be instrumental in reducing drought impacts especially in developing countries. Our results indicate that GIDMaPS data sets reliably captured several major droughts from across the globe.

## Background & Summary

A 2007 ministerial summit with representatives from 70 nations, held in Cape Town, South Africa, recognized the growing problem of drought and its impact on food security and sustainability of water resources, and highlighted the need for a global drought early warning system^1^. Drought effects are incremental and happen over a long period of time, hence receive little attention in early phases^2^.

Each year droughts result in significant socioeconomic losses and ecological damage across the globe. Given the growing population and climate change, water and food security are major challenges facing humanity^3–7^. Nearly 1 million people perished in East Africa in the mid-eighties because of a major drought that led to a widespread famine. More recently, in 2010-2011, two events in East Africa and Southeast Asia affected 9 million people, causing famine in East Africa and significant ecological impacts in Southeast Asia^8,9^. Production of adequate food to avoid food crises requires advanced drought early warning and prediction systems. In particular, a global model is needed that can support regions where famine and food crisis are prevalent because of economic and social instability and climatic variability^10^.

In recent years, several research and operational drought monitoring models have been developed. However, drought warning and prediction systems are still the least developed systems among other natural disasters primarily because of the complex processes involved in drought onset and development^2^. On a regional scale, there are a number of drought monitoring tools tailored for local to continental scale applications such as, the U.S. Agency for International Development (USAID) Famine Early Warning System Network (FEWS Net)^11^, United States Drought Monitor^12^, African Drought Monitor^13^, and the University of Washington Experimental Surface Water Monitor^14,15^.

In an interconnected world where a drought in the United States, Russia or Australia could affect global food prices^16^, a regional perspective to drought monitoring and prediction may not be sufficient. Currently, few global drought models are available, including the Global Information and Early Warning System on Food and Agriculture (GIEWS), Humanitarian Early Warning Service (HEWS) by the World Food Programme (WFP), Global Drought Monitor by the University College London (UCL), the Standardized Precipitation-Evapotranspiration Index Global Drought Monitor^17^, and the Global Drought Portal (GDP) by the United States National Climatic Data Center^18^. The aforementioned models, however, do not provide probabilistic forecasts of drought condition for risk assessment (see ref. 2 for a comprehensive review). For example, the FAO-GIEWS provides monthly briefings on countries under drought that are facing food crisis. The HEWS gathers drought information from various sources and summarizes the information into brief descriptions of the current conditions. The UCL and GDP provide interactive maps of the latest drought condition. While these outputs are valuable, probabilistic seasonal drought forecasts can substantially improve drought early warning capability.

United Nations Environment Programme (UNEP) report calls for a drought prediction system based on a comprehensive and *integrated* approach that would include multiple drought indicators^2^. While droughts originate from a deficit in precipitation, an effective drought monitoring and prediction system should integrate multiple drought-related variables^12,19–21^. The United Nations (UN) Strategy for Disaster Reduction (ISDR) argues that an early warning system should have the following features^22^: (a) Monitoring and predicting components, (b) Risk knowledge, (c) Disseminating information, and (d) Response.

Following the above recommendation, the Global Integrated Drought Monitoring and Prediction System (GIDMaPS) is developed to provide drought information based on multiple drought indicators and input data sets. GIDMaPS includes a seasonal probabilistic prediction component that supports *risk knowledge*. The probabilistic forecasts offer essential information for early warning, preventive measures, and mitigation strategies. GIDMaPS provides both monitoring and prediction components, as well as a data dissemination interface.

## Methods

### GIDMaPS drought monitoring and prediction

**Figure 1:**
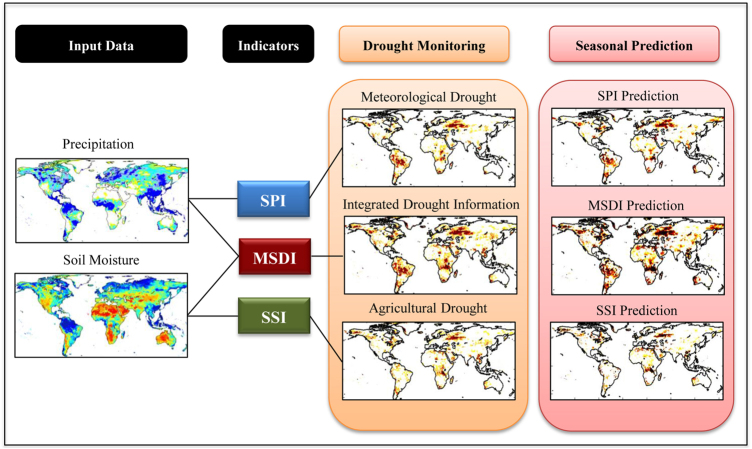
Schematic view of the GIDMaPS algorithm (SPI: Standardized Precipitation Index; SSI: Standardized Soil Moisture Index; and MSDI: Multivariate Standardized Drought Index).

The algorithm of the GIDMaPS is schematically illustrated in Figure 1. GIDMaPS integrates precipitation and soil moisture data from model simulations and remote sensing observations including the Modern-Era Retrospective analysis for Research and Applications (MERRA-Land^23,24^), North American Land Data Assimilation System (NLDAS^25,26^), Global Land Data Assimilation System (GLDAS^27^) and the Global Drought Climate Data Record (GDCDR^28^). GDCDR combines real-time Precipitation Estimation from Remotely Sensed Information using Artificial Neural Networks (PERSIANN) satellite data^29,30^ with long-term GPCP^31^ observations using a Bayesian algorithm. Table 1 summarizes the input data sets, their spatial resolutions, and providers.

GIDMaPS uses three drought indicators for monitoring and prediction: the Standardized Precipitation Index (SPI^32^), Standardized Soil Moisture Index (SSI^20^), and Multivariate Standardized Drought Index (MSDI^33^). SPI and SSI are indicators of meteorological and agricultural drought, respectively. Both SPI and SSI are derived using a nonparametric approach outlined in^33^. In this method, the marginal probability distribution of precipitation and soil moisture are computed using the empirical Gringorten plotting position^34^. In other words, instead of fitting a parametric distribution function, the probabilities of observed precipitation and soil moisture are computed empirically. The empirical probabilities are then standardized as *SPI*=ϕ^−1^(*p*_*p*_) and *SSI*=ϕ^−1^(*p*_*s*_) where ϕ is the standard normal distribution (probability density function), and *p*_*p*_ and *p*_*s*_ denote the empirical probabilities of precipitation and soil moisture, respectively.

MSDI provides a composite model based on both precipitation and soil moisture and can be considered an integrated meteorological and agricultural drought indicator. The nonparametric framework discussed above, can be extended to a multivariate form for estimation of MSDI^33^. Denoting *P* and *S* as vectors of precipitation and soil moisture data, the joint distribution of the two variables can be expressed as: *Pr*(*P*≤*p*, *S*≤*s*). Throughout this paper, uppercase characters represent random variables, and lowercase letters express their realizations. The empirical joint probability of precipitation and soil moisture *p*(*p*_*k*_, *s*_*k*_) can then be obtained using the multivariate model of the Gringorten plotting position^35^: $p(p_{k},s_{k})=\frac{m_{k}-0.44}{n+0.12}$, where *m*_*k*_ is the number of occurrences of the pair (*p*_*i*_, *s*_*i*_) for *p*_*i*_≤*p*_*k*_ and *s*_*i*_≤*s*_*k*_, and *n* is the sample size. The joint empirical probabilities of *P* and *S* can be standardized to derive the MSDI (*MSDI*=*ϕ*^−1^(*p*)).

As shown in Figure 1, GIDMaPS includes a monitoring component that provides drought information based on historical observations, and a prediction component for seasonal drought forecasting. The prediction component of GIDMaPS is based on the concept of the Ensemble Streamflow Prediction (ESP) method that has been used in numerous hydrology and climate studies^36–39^. This approach assumes that historical data from any location are possible scenarios for the future. The GIDMaPS’s prediction component extends persistence-based drought prediction to a multi-index framework based on multiple variables, providing short-term forecasts based on both univariate (SPI, SSI) and multivariate (MSDI) drought indicators.

Let's assume an n-year climatology is available and drought condition in month *m* of year *n*+1 is to be predicted. GIDMaPS utilizes 6 month accumulated precipitation (*AP*_*n*+1,*m*_) and soil moisture (*AS*_*n*+1,*m*_) for month *m* of year *n*+1 as predictants:

(1)APn+1,m=Pn+1,m-5+Pn+1,m-4+Pn+1,m-3+Pn+1,m-2+Pn+1,m-1+Pn+1,m

(2)ASn+1,m=Sn+1,m-5+Sn+1,m-4+Sn+1,m-3+Sn+1,m-2+Sn+1,m-1+Sn+1,m

Here, the terms *P*_*n*+1,*m*_ and *S*_*n*+1,*m*_ refer to precipitation and soil moisture in the target month *m* (1-month lead). The other terms are initial conditions for the target month predictions (for *m*= 1 initial conditions will be sampled from year *n*). In a persistence-based model, predictions of *P*_*n*+1,*m*_ and *S*_*n*+1,*m*_ for the target month *m* can be sampled based on *n* previous observations of precipitation and soil moisture from the climatology:

(3)APn+1,mi=Pn+1,m-5+Pn+1,m-4+Pn+1,m-3+Pn+1,m-2+Pn+1,m-1+Pi,m,i=1,…,n

(4)ASn+1,mi=Sn+1,m-5+Sn+1,m-4+Sn+1,m-3+Sn+1,m-2+Sn+1,m-1+Si,m,i=1,…,n

Having *AP*_1,*m*_,…,*AP*_*n,m*_ and *AS*_1,*m*_,…,*AS*_*n m*_ from equations 3 and 4, the MSDI^*i*^ can be computed as:

(5)MSDIi=P(AP≤APn+1,mi,AS≤ASn+1,mi),i=1,…,n

Basically, in this approach, an *n*-year climatology (historical observations) leads to an *n*-member ensemble. GIDMaPS uses the ensemble median as a measure of drought severity. Having an ensemble of predictions, GIDMaPS offers the probability of drought occurrence for any given drought threshold. For example, for a standardized index (e.g., SPI, SSI, MSDI), the occurrence probability of drought below a certain threshold can be estimated as the number of ensemble members *n*_*x*_ below the choice of threshold divided by the total number of members (*n*_*x*_/*n*). Source code is available from the figshare record associated with this publication (Data Citation 1).

## Data Records

GIDMaPS's data records are standardized drought indices in which a negative (positive) value indicates a relatively dry (wet) spell. The monitoring component of GIDMaPS provides information on drought severity in both the original standardized scale and the so-called D-scale^12^ (see Table 2). The prediction component offers probability of drought occurrence computed for different drought severity levels. For example, the prediction component provides the probability of a drought index below −0.8 or D1 drought severity.

GIDMaPS data sets are available to the public through an unrestricted repository at http://dx.doi.org/10.6084/m9.figshare.853801 (Data Citation 1) in a simple ASCII format (Longitude, Latitude, Drought Severity). These data sets provide a static representation of the data at the time of publication, as a complement to the periodically updating data distribution system available at http://drought.eng.uci.edu/. The data distribution system delivers both graphical images and raw data. Table 3 summarizes the GIDMaPS drought information outputs. The prediction data sets provide 1 to 6 month lead forecasts based on the indices shown in Table 3, and from the last monitoring data available. The system runs on a daily basis and updates the data record upon availability of new input data sets (Table 1).

### Technical Validation

**Figure 2:**
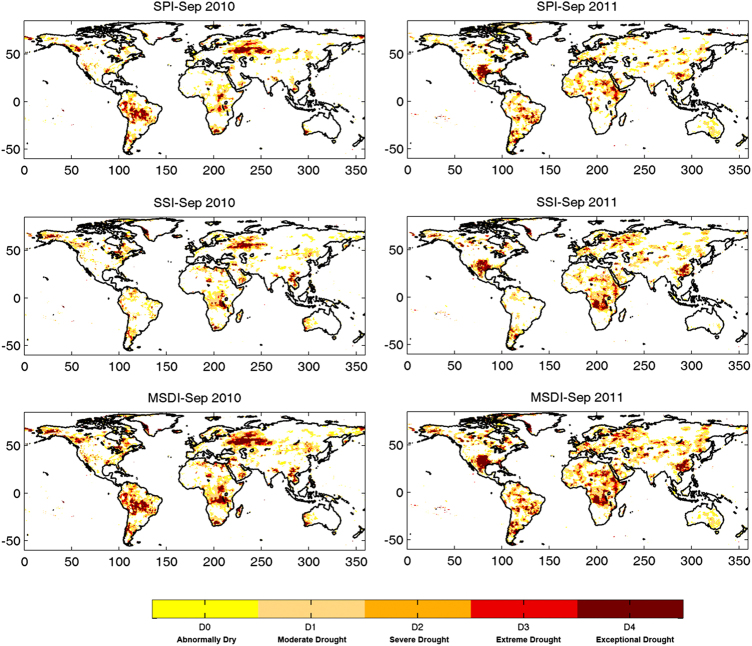
Drought monitoring based on multiple indicators: Standardized Precipitation Index (SPI; 1st row); Standardized Soil Moisture Index (SSI; 2nd row); and Multivariate Standardized Drought Index (MSDI; 3rd row). The SPI shows meteorological drought, whereas the SSI represents agricultural drought. MSDI is a composite model of both meteorological-agricultural drought conditions. Input data sets include MERRA-Land precipitation and soil moisture fields.

Sample drought monitoring outputs based on GIDMaPS indicators are presented in Figure 2. The three rows in Figure 2 display global drought information based on SPI, SSI and MSDI for 2010 and 2011. As shown, the 2010 Amazon drought^40^, 2010 Russian drought^16^, 2011 Texas-Mexico drought^41^ and 2011 East Africa drought^9^ are well captured by GIDMaPS.

**Figure 3:**
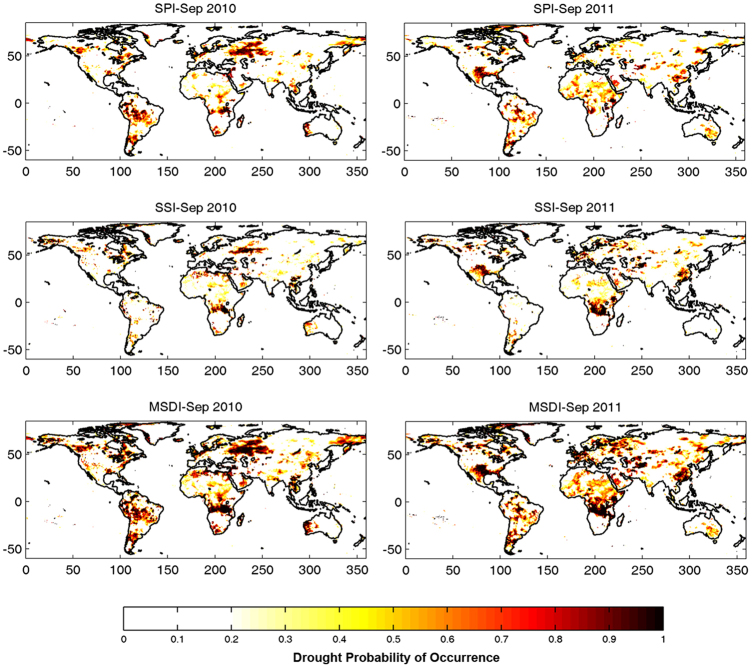
GIDMaPS 2 month lead drought prediction (probability of occurrence) based on multiple indicators: Standardized Precipitation Index (SPI; 1st row); Standardized Soil Moisture Index (SSI; 2nd row); and Multivariate Standardized Drought Index (MSDI; 3rd row). Input data sets include MERRA-Land precipitation and soil moisture fields.

For events shown in Figure 2, the 2 month lead drought predictions are presented in Figure 3. Notice that the scales of the two figures are different. The monitoring component provides drought severity, whereas the prediction component offers the probability of drought occurrence computed, as described in the Methods section. It is worth pointing out that probability of drought occurrence can be computed for different drought severity thresholds (Methods Section). Figure 3, for example, provides probability of drought occurrence for the D1 threshold, meaning the probabilities correspond to D1-D4 droughts. The results indicate that the regions where drought is predicted with high probability in Figure 3 are consistent with the observed droughts shown in Figure 2 (compare the Amazon and Russia in 2010 and Texas-Mexico and East Africa in 2011 in Figures 2 and 3).

**Figure 4:**
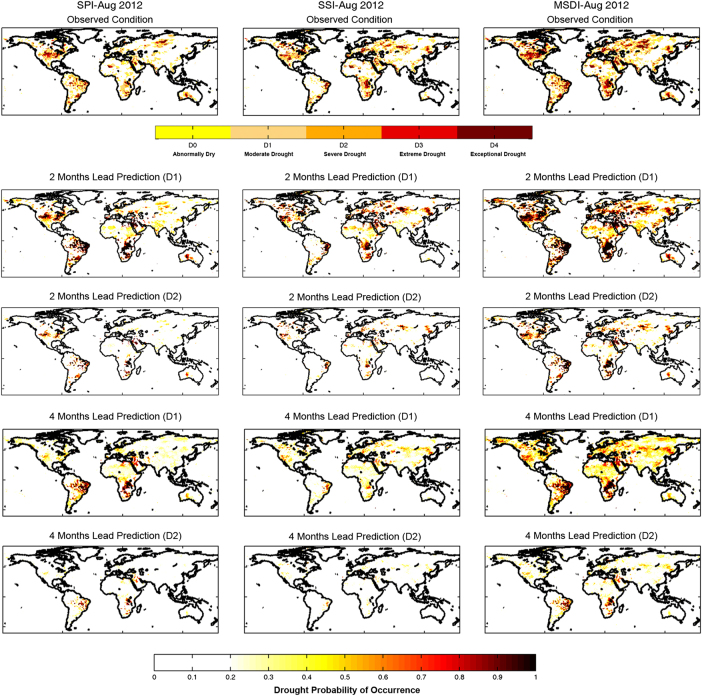
2 and 4 month lead predictions of moderate (D1) and severe (D2) drought conditions for August 2012.

GIDMaPS data sets provide drought predictions for different lead times. For August 2012, for example, Figure 4 demonstrates 2 and 4 month lead predictions of drought for D1 and D2 severity levels. In 2012, the United States experienced a major drought that led to significant losses (see the top row in Figure 4). The 2 month lead predictions identify the upcoming drought at both D1 and D2 severity levels. While the 4 month lead predictions show signals of the U.S. summer 2012 drought at the D1 level, only MSDI indicates the possibility of a D2 (or stronger) drought with 4 month lead time (probabilities ranging from 0.3 to 0.6 in the central and western United States). It is stressed that the dynamical weather and climate models initiated in April/May 2012 did not indicate a significant drought coming up in August 2012^42^. This highlights the importance of GIDMaPS seasonal forecasts, providing relatively reasonable forecasts 2 to 4 month in advance that could be very important for risk assessment and decision making.

### Usage Notes

**Figure 5:**
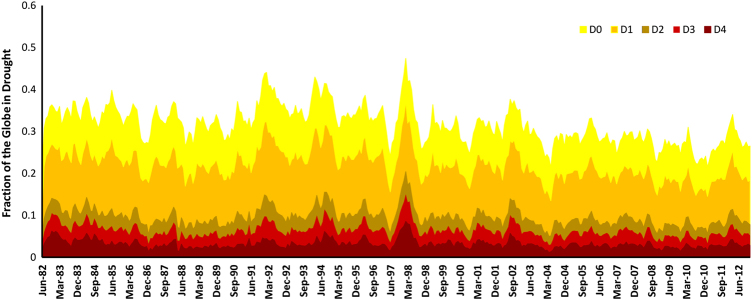
Fraction of the global land in D0 (abnormally dry), D1 (moderate), D2 (severe), D3 (extreme), and D4 (exceptional) drought condition (Data: Standardized Precipitation Index data derived from MERRA-Land).

Drought data records are fundamental to study regional/global changes to trends and patterns of droughts. GIDMaPS’s data sets listed in Table 3 can be used for a wide variety of applications/studies. For example, GIDMaPS climate data records can be used to assess the fraction of global land areas under D0 to D4 drought severity levels as displayed in Figure 5. The figure highlights a substantial increase in severe to exceptional drought in the late nineties as discussed in previous studies (e.g., see ref. 43). The figure indicates that in the peak time, around 20% of global land areas were in severe to exceptional drought, a record drought the likes of which has not been experienced since.

**Figure 6:**
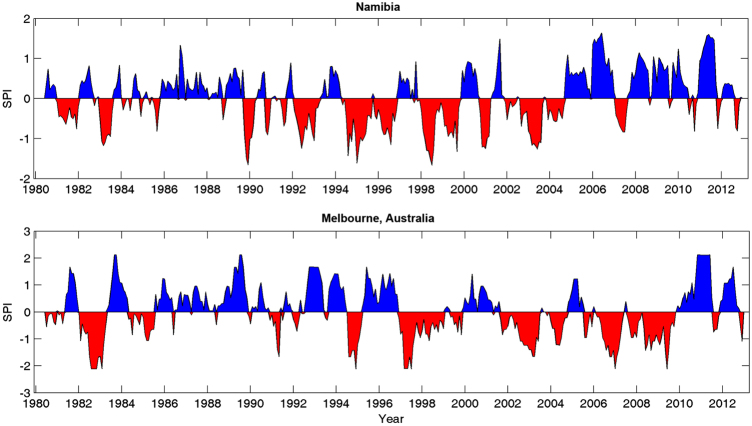
Time series of the 6 month Standardized Precipitation Index (SPI) averaged over Namibia (top) and Melbourne, Australia (bottom) between 1980–2012.

A region's drought climatology can also be investigated using GIDMaPS data sets. One can obtain the fraction of a region/country under drought and assess trends in temporal patterns of areas in drought. In a recent study, global trends and spatial patterns of droughts are investigated using GIDMaPS data^44^. Additionally, users can evaluate drought duration and severity in historical records. As an example, the time series of the 6 month SPI for Namibia and Melbourne, derived from GIDMaPS, are presented in Figure 6. The figure shows that both regions often experience multi-year dry and wet periods. This information can be used to place a specific drought, such as the Australian Millennium Drought^45^ (see 1997–2009 in Figure 6), in perspective relative to historical observations. Combined with other data sets (e.g., global temperature data), GIDMaPS data can be used to assess changes in drought and heatwaves^46^ or warm/dry, cold/dry conditions^47^.

GIDMaPS data records can also be of interest to ecologists for studying the effects of historical droughts on vegetation growth, tree mortality, and ecosystem behavior. Furthermore, water and energy resources as well as the agricultural sector are sensitive to droughts^48–50^. The 2012 United States drought, for example, resulted in over $12 billion in economic loss, and significant indirect effects on the global food prices^51^. Seasonal prediction of drought conditions can provide resource managers with valuable information for decision making and disaster preparedness.

Finally, dynamic models that provide global seasonal drought forecasts exhibit very high uncertainty and low seasonal prediction skill^52–54^. It is our vision that drought information should be based on a wide variety of sources, data, indicators, and models. GIDMaPS provides a different perspective using a statistical model extended for multivariate drought monitoring and prediction. We stress the purpose of GIDMaPS is not to replace the currently available dynamic models. Rather, GIDMaPS data sets should be used as additional source of drought information to improve current capabilities in drought monitoring and prediction.

## Additional information

**How to cite this article:** Hao, Z. *et al.* Global integrated drought monitoring and prediction system. *Sci. Data* 1:140001 doi: 10.1038/sdata.2014.1 (2014).

## Supplementary Material



## Figures and Tables

**Table 1:** GIDMaPS's input data sets. GDCDR: Global Drought Climate Data Record^28^; GLDAS: Global Land Data Assimilation System^27^; GPCP: Global Precipitation Climatology Project^55^; MERRA: NASA Modern-Era Retrospective analysis for Research and Applications^23^; NLDAS: North American Land Data Assimilation System^25,26^; UCI: University of California, Irvine.

**Input data**	**Variables**	**Source**	**Spatio-temporal resolution**
MERRA-Land	Precipitation and soil moisture	NASA	2/3°×1/2°, monthly
NLDAS	Precipitation and soil moisture	NASA	0.125°, monthly
GDCDR	Precipitation	UCI, GPCP	0.5° & 2.5°, monthly
GLDAS	Precipitation and soil moisture	NASA	1°, monthly

**Table 2:** Drought severity information in both the original standardized scale and their corresponding D-scale.

**Standardized index**	**D-scale**	**Description**
−0.50 to −0.79	D0	Abnormally dry
−0.80 to −1.29	D1	Moderate drought
−1.30 to −1.59	D2	Severe drought
−1.60 to −1.99	D3	Extreme drought
−2.0 or less	D4	Exceptional drought

**Table 3:** GIDMaPS's output data records. GDCDR: Global Drought Climate Data Record; GLDAS: Global Land Data Assimilation System; MERRA: NASA Modern-Era Retrospective analysis for Research and Applications; MSDI: Multivariate Standardized Drought Index; NLDAS: North American Land Data Assimilation System; SPI: Standardized Precipitation Index; SSI: Standardized Soil Moisture Index.

**Data records**	**Availability**	**Resolution**
MERRA-based SPI, SSI and MSDI	1980-present	2/3°×1/2°
NLDAS-based SPI, SSI and MSDI	1979-present	0.125°
GDCDR-based SPI	1980-present	0.5° & 2.5°
GLDAS-based SPI, SSI and MSDI	1949-present	1°
